# Feasibility and efficacy of ‘Can-Sleep’: effects of a stepped-care approach to cognitive-behavioral therapy for insomnia in cancer

**DOI:** 10.1007/s11764-023-01457-3

**Published:** 2023-09-26

**Authors:** Justine Diggens, Dani Bullen, Jordan Maccora, Joshua F. Wiley, Steve Ellen, Jeremy Goldin, Michael Jefford, Martha Hickey, Maria Ftanou

**Affiliations:** 1 Peter MacCallum Cancer Centre, 3000 Melbourne, VIC Australia; 2 School of Psychological Sciences and Turner Institute for Brain and Mental Health, Monash University, Melbourne, Australia; 3 Melbourne School of Population and Global Health, University of Melbourne, Melbourne, Australia; 4 Department of Health Services Research, Peter MacCallum Cancer Centre, Melbourne, Australia; 5 Australian Cancer Survivorship Centre, Peter MacCallum Cancer Centre, Melbourne, Australia; 6 Sir Peter MacCallum Department of Oncology, University of Melbourne, Melbourne, Australia; 7 Department of Obstetrics and Gynaecology, University of Melbourne and the Royal Women’s Hospital, Melbourne, VIC Australia

**Keywords:** Cancer, Insomnia, Sleep, Cognitive behavioral therapy, Stepped-care

## Abstract

**Purpose:**

This study aimed to evaluate the feasibility and clinical efficacy of the Can-Sleep stepped-care intervention for people with cancer-related sleep disturbance.

**Methods:**

A total of 147 individuals with cancer were screened. Participants who reported sleep disturbances and were at low-moderate risk for intrinsic sleep abnormalities were given self-managed cognitive behavioral therapy for insomnia (SMCBT-I). Those reporting sleep disturbance and scoring at high risk of intrinsic sleep abnormalities (i.e., restless leg syndrome and obstructive sleep apnoea) were referred to a specialist sleep clinic. In both groups, participants received a stepped-up group CBT-I intervention (GCBT-I) if they continued to report sleep disturbance following SMCBT-I or the specialist sleep clinic.

**Results:**

Overall, 87 participants reported sleep disturbance or screened at risk for intrinsic sleep abnormality. Thirty-four were referred to a specialist sleep clinic, and of the 17 who declined this referral, 14 were rereferred to SMCBT-I. In total, 62 participants were referred to SMCBT-I, and 56 commenced SMCBT-I. At post-intervention, the SMCBT-I group showed a significant decline in insomnia symptoms (*p* < .001, *d* = 1.01). Five participants who reported sleep disturbance after SMCBT-I and/or the specialist sleep clinic, accepted GCBT-I. Those who received the GCBT-I showed a significant reduction in insomnia symptoms (*p* < .01, *d* = 3.13).

**Conclusions:**

This study demonstrates the feasibility and efficacy of a stepped-care intervention for sleep disturbances in people with cancer.

**Implications for cancer survivors:**

A stepped-care intervention for sleep disturbance is a feasible and potentially effective method of addressing a significant and unmet patient need.

## Introduction

In high-income countries, over one-third of the population are estimated to receive a cancer diagnosis in their lifetime [1, 2]. In Australia, over 160,000 new cancer diagnoses are estimated in 2022, with 68.9% of people expected to survive at least 5-years, resulting in a significant portion of the population either having cancer or living with the aftermath of cancer [2]. Sleep disturbance disproportionately affects people with cancer [3, 4] due to the physical and psychological sequelae of cancer diagnosis and associated anti-cancer treatment. In a recent population-based survey (*N =* 28,159), cancer survivors reported sleep disturbance (34%), with prevalence of approximately 50% higher than the general population with no history of cancer (23%) [3]. Further, longitudinal data from a sample of women identifying as ‘good sleepers’ prior to breast cancer diagnosis (*N =* 73), revealed 77% met the criteria for insomnia syndrome at least once within the 12-months after diagnosis [5]. Notably, cancer-related sleep disturbance may persist, with 30–60% of cancer survivors reporting sleep complaints up to five years posttreatment [4, 6–8].

Sleep disturbance has wide-reaching consequences. At an individual level, insomnia is associated with anxiety [9], depression [10], concentration and memory difficulties, lower quality of life [11–13], higher rates of pain [14], increased use of sedatives [15] and poorer health outcomes [16, 17]. The burden of sleep disturbance extends beyond these personal costs to a wider economic and social impact on the broader community. In cancer survivors, sleep partially accounts for the impact of cancer on healthcare expenditures and work absenteeism [18]. Nationally, the estimated costs of inadequate sleep in Australia in 2016–2017 were $66.3 billion, including health system costs, productivity losses, information care costs, other financial costs, and wellbeing costs [19].

Cognitive behavior therapy for insomnia (CBT-I) is a non-pharmacological approach that is considered the gold-standard treatment for insomnia [20]. CBT-I is a multicomponent intervention that targets dysfunctional beliefs and behaviors that perpetuate sleep disturbance using sleep hygiene, sleep restriction, stimulus control, cognitive restructuring, and relaxation strategies. Meta-analyses of CBT-I in cancer support its efficacy in reducing insomnia and fatigue symptoms [21, 22]. CBT-I has also shown beneficial effects on menopausal symptoms [23], mood [24, 25], cognitive functioning [26], quality of life [27], immunological function and need for medication [28]. The effects of CBT-I may be durable, with randomised controlled trials (RCT) showing benefits lasting up to three years post CBT-I treatment [29, 30] and meta-analyses showing benefits through 6-month follow-up [21, 22]. Research also suggests CBT-I has superior effects on insomnia (more consistent, rapid and durable in its improvements) compared to Mindfulness Based Stress Reduction (MBSR) [31], pharmacotherapies [32, 33] and acupuncture [34]. Up to 77% of individuals with cancer have been found to achieve remission or significant reduction of insomnia symptoms after face-to-face CBT-I [35]. Lastly, CBT-I has been found to be more cost-effective than pharmacological treatment of insomnia [36].

Despite its established efficacy, many individuals with cancer do not access CBT-I. There are several reasons for this, including lack of early identification, limited availability of specialised CBT-I services and the relatively high cost of individual sessions [37]. The prevalence of sleep disturbance in cancer [4], combined with projections of new cancer diagnoses in Australia in 2022 [2], suggest approximately 55,000 new people with cancer will experience sleep disturbance in 2022 in Australia alone, supporting the critical need for increased access to effective treatments.

A stepped-care approach may represent a suitable option to addressing barriers faced in an oncology setting. Stepped-care approaches commence with low intensity and low resources interventions and steps people up to more intense interventions based on symptom severity or if symptoms progress or do not improve with lower level interventions. Stepped-care reduces delivery costs whilst increasing accessibility by systematically increasing treatment potency across ‘steps’. For example, in the first instance, low-intensity and scalable interventions (e.g., self-management CBT-I) are disseminated to all reporting insomnia symptoms, whereas, intensive “stepped-up” care (e.g., group CBT-I) is offered when symptoms continue after receiving the initial intervention step [38].

A growing body of literature has investigated CBT-I across different modalities. Meta-analyses show that self-management CBT-I resources are effective for treating insomnia in the general population, especially when paired with therapist support during the program [39, 40]. Although limited, existing research suggests that self-management CBT-I is also effective for people with cancer [41]. More intensive treatment modalities have also shown effectiveness. One meta-analysis has shown that group CBT-I is effective at reducing insomnia symptoms in the general population [42]. One feasibility study in 11 people showed adequate attendance (67% of sessions) and satisfaction (57.1%) with a 9-week group delivered CBT-I for sleep intervention in breast cancer survivors [43]. A second study in 51 cancer survivors showed efficacy for a stepped care model where the first step was a single sleep education session, and the second step was group-delivered CBT-I [44].

Treating sleep disturbance in cancer is critical due to the growing number of people affected and the significant burden it imposes on their lives. However, it remains unclear what the best model is to deliver CBT-I to a diverse cancer population. Therefore, it is imperative to prioritize the development and evaluation of resource-efficient and accessible approaches to address this issue. The primary objective of this study was to assess the feasibility and effectiveness of a stepped-care approach for managing sleep disturbance in individuals with cancer.

## Method

### Design

**Fig. 1:**
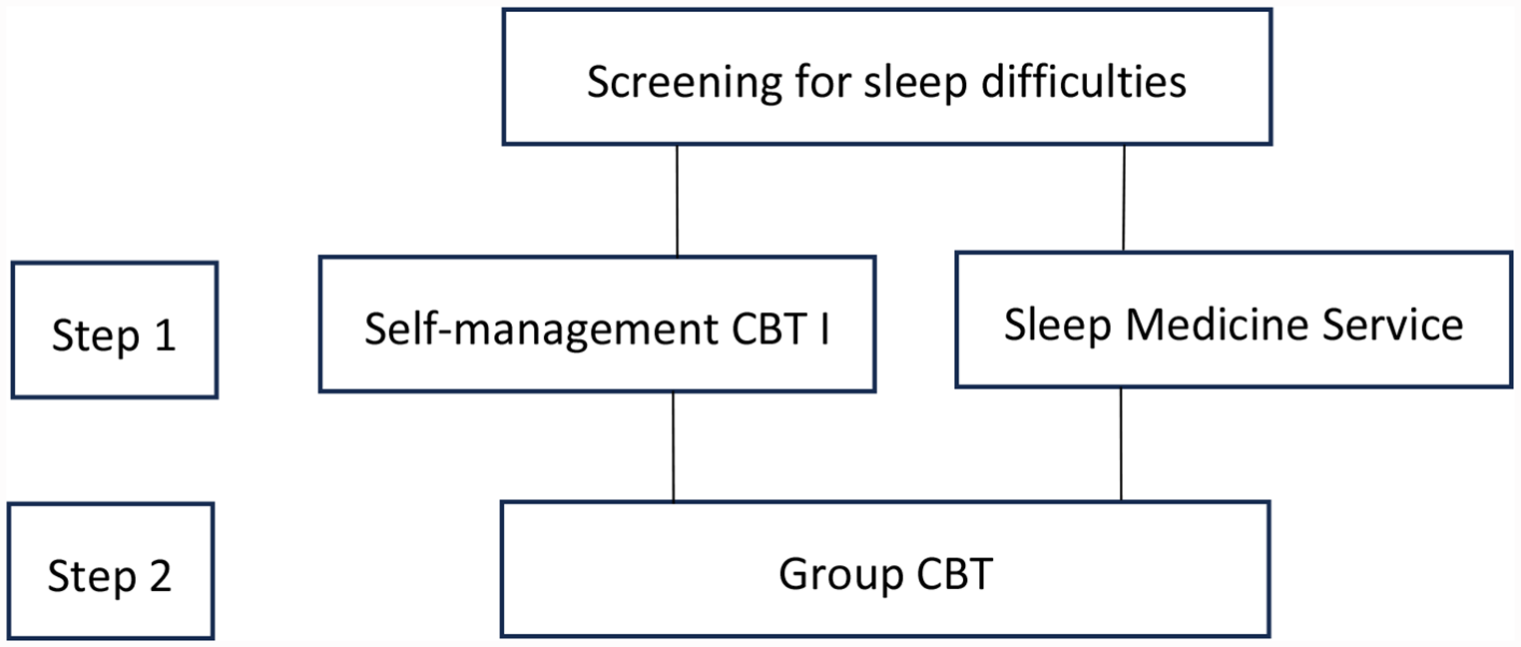
Can-sleep interventions

This study utilized a prospective, single-arm design to evaluate the feasibility and utility of the Can-Sleep stepped-care CBT-I intervention for cancer-related sleep disturbance. As described in Fig. 1 Can-Sleep included two intervention steps. The entry level of the Can-Sleep intervention differed depending on baseline risk of restless leg syndrome (RLS) and obstructive sleep apnoea (OSA): those at high-risk of either condition were referred to a Sleep Medicine Service, while those at lower risk were offered self-managed CBT-I. The second step, offered to participants who continued to endorse elevated insomnia severity, consisted of four face-to-face group CBT-I sessions delivered weekly or fortnightly. Study measures were collected at pretreatment (T1, week 0), immediately after Step 1 of the intervention (T2, week 5), and Step 2 of the intervention (T3, week 9). The protocol received approval from the Peter MacCallum Cancer Centre Human Research Ethics committee (17/83L).

### Participants

Between November 2017 and March 2018, participants were recruited from three metropolitan hospitals and six outpatient oncology clinics (breast, gynae-oncology, lung, late effects, genitourinary or haematology) in Melbourne, Australia.

Inclusion criteria were English-speaking people aged > 25 who had received a cancer diagnosis across their lifespan. People under the age of 25 were excluded because they receive services at adolescent and young adult cancer service which is a separate service. They may also have a range of different development factors impacting their sleep. No restrictions were placed on time since initial diagnosis, cancer stage, or presence/absence of anti-cancer treatment at enrolment.

### Measures

*Insomnia Severity Index* (ISI) (44). The ISI is a seven-item self-report measure that assess insomnia severity over the previous two weeks. Responses are provided on a 5-point Likert scale (0 = *“None”*, 4 = *“Very Severe”*), with higher scores indicating increased insomnia severity. Total scores range from 0 to 28. A cut off of > 7 was used to identify sleep disturbance based on established thresholds.

*Epworth Sleepiness Scale* (ESS) [45]. The ESS is a self-report questionnaire that assesses daytime sleepiness across eight common situations. Responses range from 0 (*“would never doze”*) to 3 (“*high chance of dozing*”). Total scores range from 0 to 24. Scores > 10 were interpreted as clinically significant excessive daytime sleepiness.

*STOP-BANG* [46]. The STOP-BANG is an eight-item self-report questionnaire that assesses OSA risk factors. Items are dichotomous (*“yes”, “no”)* and total scores range from 0 to 8, with higher scores indicating higher risk of OSA. Total scores > 4 was classified as high risk of moderate to severe OSA. A total score of 3 or 4, was also deemed as high risk if participants had a BMI > 35 kg/m^3^ or a neck circumference ≥ 43 cm in males or ≥ 41 cm in females at the time of screening. STOP-Bang has demonstrated adequate sensitivity [47].

*Restless Leg Screening Tool* (RLST). The RLST is a purpose-built self-report measure comprising five dichotomous items (*“yes”, “no”)* which reflect the diagnostic criteria for RLS in DSM-V. Participants who endorsed all five items as “*yes*” were considered at high risk of RLS.

### Procedure

Participants who were eligible and had provided informed consent were screened using the ISI and ESS. Participants who met the criteria (> 7 ISI and/or > 10 ESS) were asked to complete screening measures for OSA (STOP-BANG) and RLS (RLST), and subsequently offered the Can-Sleep stepped-care intervention. Step 1 of the intervention differed depending on baseline risk of OSA and RLS.

### Intervention

*Step 1: participants at high risk of OSA and/or RLS at T1.* Participants who scored high on the STOP-BANG (> 4) or the RLST (i.e., yes to all questions) were referred to the Sleep Medicine Service at a participating hospital for further assessment. Participants who scored below cutoffs on both the STOP-BANG and RLSS, or declined the Sleep Medicine Service were offered self-managed CBT-I.

*Step 1: participants at low-moderate risk of OSA and/or RLS at T1.* Participants were provided with a 5-week CBT-I self-management resource (SMCBT-I). SMCBT-I comprised a 23-page colour printed booklet to improve sleep in individuals with cancer. The booklet was developed with the input of approximately 60 cancer specialists, sleep physicians, psychologists, a GP, psychiatrist, gynaecologist and cancer consumers. The booklet included the following:

- Psychoeducation about sleep and causes of poor sleep including: stages of sleep; sleep drive; circadian rhythm; sleep deprivation; and acute and chronic insomnia.
- Key cognitive and behavioral methods to address night-time sleep disturbances or insomnia in cancer, including: sleep hygiene strategies, developing helpful sleep cognitions; stimulus control; sleep restriction; strategies to manage stress or worry (including relaxation and worry postponement); relapse prevention and information on medications and other useful services and resources in the public domain.

This booklet included additional content tailored to individuals with cancer, including:

- Illustrations and examples of common worries and cognitions in people with cancer that interfere with sleep.
- Strategies to manage common cancer-related problems (e.g., hospitalisation) and cancer-treatment symptoms and side-effects that impact sleep (i.e.,nausea, hot flushes), and mention of sleep strategies that may be more challenging for people with cancer (e.g., deep breathing when experiencing shortness of breath).
- Content on how to manage and think about pain and unpleasant sensations at night-time (e.g., nausea, hot flushes, rash, symptoms of peripheral neuropathy) commonly caused by cancer, cancer treatment or side-effects.

Participants received two supporting consultations with a trained member of the clinical team: (1) at the beginning of the intervention, participants received instruction on how to effectively use the SMCBT-I resource; and (2) approximately three weeks after receiving the SMCBT-I resource, participants were phoned to assist with strategy implementation, answer queries and encourage adherence.

Participants were rescreened via phone at the conclusion of Step 1 (T2). Remitted participants (ISI < 8), or participants endorsing subclinical insomnia severity (ISI > 7 and < 15) who were satisfied with sleep improvements following Step 1 received no further treatment. Participants who continued to endorse sleep complaints (ISI > 7) progressed to Step 2.

*Step 2: participants having an ISI score > 7 at T2.* Participants received four 90-minute, face-to-face CBT-I group sessions (GCBT-I) facilitated by two clinical psychologists. The content of these sessions included core CBT-I principles, whilst also addressing symptoms and side-effects unique to individuals with cancer. Recommendations were individualized according to presenting concerns and subjective sleep diary reports (e.g., sleep restriction time-in-bed). Participants who declined the GCBT-I program had alternative treatment options discussed with them (e.g., individual CBT-I sessions). Participants were rescreened using the ISI in the week following the completion of the GCBT-I sessions.

### Statistical analysis

Descriptive statistics are frequencies and percentages for discrete variables and means and standard deviations for continuous variables. Change in insomnia symptoms from pretreatment to posttreatment was tested using paired t-tests. Significance was set at two-tailed α = 0.05.

## Results

### Sample characteristics

**Fig. 2:**
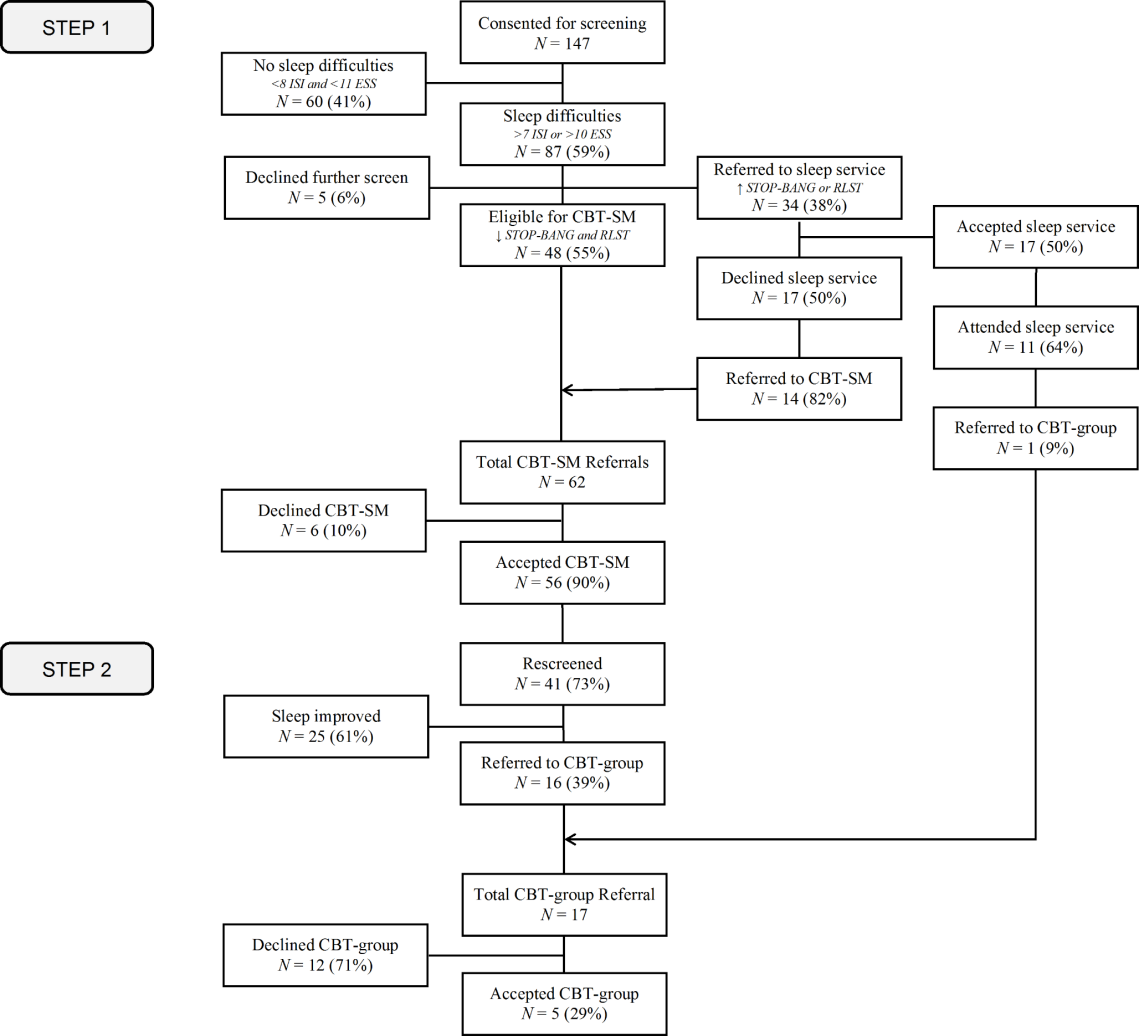
Participant Flow Chart *Note*. STOP-BANG, assessed obstructive sleep apnoea; RLST = Restless Leg Screening Tool, assessed restless leg syndrome; ISI = Insomnia severity index; ESS = Epworth sleepiness scale; SMCBT-I = cognitive behavioral therapy self management; GCBT-I = cognitive behavioral therapy group.

Table 1 shows the demographic and clinical characteristics of the sample. One hundred and forty-seven individuals (Age: *M* = 58.24, *SD* = 12.42) with cancer consented to participate in screening (*see* Fig. 2). The sample was primarily female (*N* = 106, 72%), comprised of individuals from the gynaecological (*N* = 47, 32%), breast (*N* = 41, 28%), haematology (*N* = 35, 24%), genitourinary (*N* = 15, 10%), lung (*N* = 7, 5%), and late effects (*N* = 2, 1%) clinics. Of the 147 participants who completed the initial screening, 87 (59%) reported sleep difficulties (ISI > 7 and/or ESS > 10) and were invited to complete additional screening (OSA/RLS). Five participants (6%) declined further screening and were withdrawn from the study.

**Table 1:** Social-demographic and clinical characteristics of the screened population

	Sleep difficulty *N* = 87				No sleep difficulty *N* = 60	Total *N* = 147
	Sleep disturbance *N =* 48	High risk OSA/RLS *N =* 34	Declined OSA/RSL screening *N =* 5	Total *N =* 87		
Age (range: 28-83y), *M* (*SD*)	52.75 (14.03)	57.85 (10.23)	63.4 (5.46)	55.36 (12.62)	62.42 (10.95)	58.24 (12.42)
Sex: Female, *N* (%)	39 (81)	25 (74)	4 (80)	68 (78)	38 (63)	106 (72)
Cancer Type, *N* (%)						
Gynae-oncology	17 (35)	9 (26)	3 (60)	29 (33)	18 (30)	47 (32)
Breast	15 (31)	11 (32)	1 (20)	27 (31)	14 (23)	41 (28)
Haematology	10 (21)	7 (21)	0 (0)	17 (20)	18 (30)	35 (24)
Genitourinary	5 (10)	3 (9)	1 (20)	9 (10)	6 (10)	15 (10)
Lung	1 (2)	4 (12)	0 (0)	5 (6)	2 (3)	7 (5)
Late effects	0 (0)	0 (0)	0 (0)	0 (0)	2 (3)	2 (1)
ISI, *M* (*SD*)	14.54 (3.98)	15.03 (5.08)	10.6 (1.67)	14.51 (4.44)	3.72 (2.15)	10.1 (6.46)
Range	8–22	3–28	8–12	3–28	0–7	0–28
ESS, *M* (*SD*)	7.65 (4.26)	8.29 (4.32)	8.6 (3.05)	7.95 (4.20)	4.05 (2.53)	6.36 (4.08)
Range	0–20	1–17	7–14	0–20	0–9	0–20

*Note*. OSA = Obstructive sleep apnoea; RLS = Restless leg syndrome; ISI = Insomnia severity index; ESS = Epworth sleepiness scale

### Step 1: SMCBT-I and/or sleep medicine service

Of the 82 participants who completed secondary screening, 34 (38%) scored at risk of OSA or RLS and were offered referral to a Sleep Medicine Service.

*Assessment by Sleep Medicine Service.* Seventeen of 34 (50%) accepted the referral and 11 (64%) attended the service. Eight participants received a diagnosis of OSA, two a diagnosis of RLS, and one did not receive a sleep diagnosis. Seventeen participants (50%) declined the Sleep Medicine Service referral and were offered the SMCBT-I intervention arm. The most common reason for declining was that participants felt that they were too busy to commit to the assessment/treatment process at the sleep service.

*SMCBT-I.* Sixty-two participants in total were offered the Can-Sleep stepped-care SMCBT-I intervention. Fifty-six (90%) participants commenced SMCBT-I. Most common reason for declining the SMCBT_I was program was that participants did not think it was needed. Of those who started SMCBT-I, 47 (84%) participants completed the 3-week follow-up call, and 41 (73%) completed posttreatment rescreening.

*ISI at post SMCBT-I (T2).* After initial treatment (T2), 18 (44%) SMCBT-I participants no longer met the criteria for sleep disturbance (ISI < 8). Thirteen (32%) participants endorsed subclinical insomnia severity (ISI > 7 and ISI < 15), seven (17%) of which reported feeling satisfied with sleep improvements, with no need for further intervention. Results showed a significant time effect on the ISI from baseline (T1; *M* = 15.15, *SD* = 3.83) to posttreatment (T2; *M* = 9.93, *SD* = 6.19), *t*(40) = 6.65, *p* < .0001, *d* = 1.01.

### Step 2: GCBT-I

Following Step 1, 17 participants (16 SMCBT-I, 1 Sleep Medicine Service) continued to report insomnia symptoms and were offered the GCBT-I intervention. Five (29%) of them accepted and completed GCBT-I. Common reasons for declining were other health priorities, not enough time and seeking individual support.

*ISI at post GCBT-I (T3).* At T3, four (80%) no longer met the criteria for sleep disturbance (ISI < 8), while one (20%) reported subclinical insomnia severity (ISI > 7 and ISI < 15). The difference between pre-treatment (T2; *M* = 17.4, *SD* = 3.13) and post-treatment (T3; *M* = 6.2, *SD* = 3.96) was significant, *t*(4) = 4.89, *p* < .01, *d* = 3.13.

## Discussion

The Can-Sleep stepped-care approach showed efficacy in reducing insomnia symptoms in cancer survivors. Almost all survivors who were offered the SMCBT-I intervention participated, and about half of them showed improvement with the SMCBT-I alone. Common barriers to accessing CBT-I, such as lack of time and concerns about stigma, may be addressed via the self-directed nature of the SMCBT-I intervention. Requiring approximately 25 min of staff time, SMCBT-I allowed survivors to work through the program at their own pace and in the comfort of their own homes, with minimal support from psycho-oncologists.

The results suggest that an intensive CBT-I group intervention can have low uptake. Despite offering the intervention to 17 survivors, only 5 accepted and completed it. This is not uncommon as in-person psychotherapeutic interventions for cancer survivors often face low uptake due to various barriers such as timing, side effects of treatments, fatigue and suitability of scheduling [48]. These findings suggest that individual sessions might be a more appropriate format for CBT-I for cancer survivors. We recently adapted Can-Sleep for adolescent and young adult cancer survivors and used individual sessions instead of group sessions, resulting in higher uptake [49]. Despite the limited uptake of group CBT-I, the outcomes for those who attended the group were positive.

Our findings are consistent with previous research supporting the efficacy of low-intensity CBT-I, both in the general population [39] and within cancer survivors [50, 51]. Accordingly, stepped-care interventions that commence with self-management may reduce demand on clinician resources and facilitate the provision of sleep support to a higher proportion of cancer survivors as a standard practice. Taken together, these results position a stepped-care intervention as a potentially effective and scalable intervention towards improving sleep in cancer survivors.

Despite clear strengths, results of the present study should be interpreted in light of several limitations. Firstly, while the study was able to capture a broad sample of survivors, some tumour streams were underrepresented in the current sample (e.g., late effects) which may limit the generalizability of findings. Secondly, the results of self-report measures need to be interpreted with caution given the potential influence of social desirability reporting by survivors and health care professionals. Thirdly there were some cancer types that appeared to have greater incidents of sleep difficulties such as breast cancer and gynaecological cancer compared to haematological cancer, the study small sample size and the potential reasons potential reasons for these differences was not explored, Future studies could further explore this and to help address sleep needs of specific cancer groups. Finally, whilst the current results provided evidence for Can-Sleep’s efficacy, future studies utilizing a randomized design and control condition is required to confirm its effectiveness. However, CBT-I has previously shown strong efficacy compared to control conditions [21] and the aim of evaluating if the delivery of these interventions were feasible was achieved.

## Conclusions

The Can-Sleep program is the first known program in Australia to systematically screen for and treat sleep disturbances in across a broad range of cancer survivors. The Can-Sleep program delivered best practice care by promoting non-pharmacological, evidence-based, low intensity first-line treatment of night-time sleep problems to cancer survivors. The stepped-care model proved to be acceptable to survivors and health professionals alike, and continued implementation of this model of care appears feasible. This provides cancer survivors with an evidence-based, low-burden treatment of sleep disturbances that requires limited specialist psychology services addressing a significant previously unmet need in cancer survivors.

## References

[1] Siegel RL, Miller KD, Wagle NS, Jemal A. Cancer statistics, 2023. Cancer J Clin. 2023;73:17–48. 10.3322/caac.21763 10.3322/caac.21763 36633525

[2] Australian Institute of Health and Welfare. Cancer data in Australia. Australian Institute of Health and Welfare; 2022. https://www.aihw.gov.au/reports/cancer/cancer-data-in-australia. (accessed November 25, 2021).

[3] Slade AN, Waters MR, Serrano NA. Long-term sleep disturbance and prescription sleep aid use among cancer survivors in the United States. Support Care Cancer. 2020;28:551–60. 10.1007/s00520-019-04849-3 31081525 10.1007/s00520-019-04849-3

[4] Palesh OG, Roscoe JA, Mustian KM, Roth T, Savard J, Ancoli-Israel S, et al. Prevalence, demographics, and Psychological Associations of Sleep disruption in patients with Cancer: University of Rochester Cancer Center–Community Clinical Oncology Program. J Clin Oncol. 2010;28:292–8. 10.1200/JCO.2009.22.5011 19933917 10.1200/JCO.2009.22.5011 PMC2815717

[5] Fleming L, Randell K, Stewart E, Espie CA, Morrison DS, Lawless C, et al. Insomnia in breast cancer: a prospective observational study. Sleep. 2019;42:zsy245. 10.1093/sleep/zsy245 30521041 10.1093/sleep/zsy245

[6] Strollo SE, Fallon EA, Gapstur SM, Smith TG. Cancer-related problems, sleep quality, and sleep disturbance among long-term cancer survivors at 9-years post diagnosis. Sleep Med. 2020;65:177–85. 10.1016/j.sleep.2019.10.008 32029206 10.1016/j.sleep.2019.10.008

[7] Savard J, Ivers H, Villa J, Caplette-Gingras A, Morin C. Natural course of Insomnia Comorbid with Cancer: an 18-Month Longitudinal Study. J Clin Oncology: Official J Am Soc Clin Oncol. 2011;29:3580–6. 10.1200/JCO.2010.33.2247 10.1200/JCO.2010.33.2247 21825267

[8] Jefford M, Ward AC, Lisy K, Lacey K, Emery JD, Glaser AW, et al. Patient-reported outcomes in cancer survivors: a population-wide cross-sectional study. Support Care Cancer. 2017;25:3171–9. 10.1007/s00520-017-3725-5 28434095 10.1007/s00520-017-3725-5

[9] Savard J, Ivers H, Savard M-H, Morin CM. Long-Term Effects of two formats of cognitive behavioral therapy for Insomnia Comorbid with breast Cancer. Sleep. 2016;39:813–23. 10.5665/sleep.5634 26715229 10.5665/sleep.5634 PMC4791615

[10] Vargas I, Perlis ML. Insomnia and depression: clinical associations and possible mechanistic links. Curr Opin Psychol. 2020;34:95–9. 10.1016/j.copsyc.2019.11.004 31846870 10.1016/j.copsyc.2019.11.004 PMC12172691

[11] Fortner BV, Stepanski EJ, Wang SC, Kasprowicz S, Durrence HH. Sleep and quality of life in breast Cancer patients. J Pain Symptom Manag. 2002;24:471–80. 10.1016/S0885-3924(02)00500-6 10.1016/s0885-3924(02)00500-6 12547047

[12] Mormont M-C, Waterhouse J. Contribution of the rest–activity circadian rhythm to quality of life in cancer patients. Chronobiol Int. 2002;19:313–23. 10.1081/CBI-120002606 11962684 10.1081/cbi-120002606

[13] Liu L, Fiorentino L, Rissling M, Natarajan L, Parker BA, Dimsdale JE, et al. Decreased health-related quality of life in women with breast Cancer is Associated with Poor Sleep. Behav Sleep Med. 2013;11:189–206. 10.1080/15402002.2012.660589 23205513 10.1080/15402002.2012.660589 PMC3594390

[14] Rumble ME, Keefe FJ, Edinger JD, Affleck G, Marcom PK, Shaw HS. Contribution of Cancer symptoms, dysfunctional sleep related thoughts, and Sleep Inhibitory Behaviors to the Insomnia process in breast Cancer survivors: a daily process analysis. Sleep. 2010;33:1501–9. 10.1093/sleep/33.11.1501 21102992 10.1093/sleep/33.11.1501 PMC2954700

[15] Daley M, Morin CM, LeBlanc M, Grégoire JP, Savard J, Baillargeon L. Insomnia and its relationship to health-care utilization, work absenteeism, productivity and accidents. Sleep Med. 2009;10:427–38. 10.1016/j.sleep.2008.04.005 18753000 10.1016/j.sleep.2008.04.005

[16] Mormont M-C, Waterhouse J, Bleuzen P, Giacchetti S, Jami A, Bogdan A, et al. Marked 24-h Rest/Activity Rhythms are Associated with Better Quality of Life, Better Response, and longer survival in patients with metastatic colorectal Cancer and good performance status. Clin Cancer Res. 2000;6:3038–45. 10955782

[17] Sandadi S, Frasure HE, Broderick MJ, Waggoner SE, Miller JA, von Gruenigen VE. The effect of sleep disturbance on quality of life in women with ovarian cancer. Gynecol Oncol. 2011;123:351–5. 10.1016/j.ygyno.2011.07.028 21855973 10.1016/j.ygyno.2011.07.028

[18] Gonzalez BD, Grandner MA, Caminiti CB, Hui SA. Cancer survivors in the workplace: sleep disturbance mediates the impact of cancer on healthcare expenditures and work absenteeism. Support Care Cancer. 2018;26:4049–55. 10.1007/s00520-018-4272-4 29869719 10.1007/s00520-018-4272-4 PMC6204101

[19] Morin CM, Benca R. Chronic insomnia. The Lancet. 2012;379:1129–41. 10.1016/S0140-6736(11)60750-2 10.1016/S0140-6736(11)60750-2 22265700

[20] Qaseem A, Kansagara D, Forciea MA, Cooke M, Denberg TD. Management of chronic insomnia disorder in adults: a clinical practice Guideline from the American College of Physicians. Ann Intern Med. 2016;165:125–33. 10.7326/M15-2175 27136449 10.7326/M15-2175

[21] Squires LR, Rash JA, Fawcett J, Garland SN. Systematic review and meta-analysis of cognitive-behavioural therapy for insomnia on subjective and actigraphy-measured sleep and comorbid symptoms in cancer survivors. Sleep Med Rev. 2022;63:101615. 10.1016/j.smrv.2022.101615 35303692 10.1016/j.smrv.2022.101615

[22] Johnson JA, Rash JA, Campbell TS, Savard J, Gehrman PR, Perlis M, et al. A systematic review and meta-analysis of randomized controlled trials of cognitive behavior therapy for insomnia (CBT-I) in cancer survivors. Sleep Med Rev. 2016;27:20–8. 10.1016/j.smrv.2015.07.001 26434673 10.1016/j.smrv.2015.07.001

[23] Mann E, Smith MJ, Hellier J, Balabanovic JA, Hamed H, Grunfeld EA, et al. Cognitive behavioural treatment for women who have menopausal symptoms after breast cancer treatment (MENOS 1): a randomised controlled trial. Lancet Oncol. 2012;13:309–18. 10.1016/S1470-2045(11)70364-3 22340966 10.1016/S1470-2045(11)70364-3 PMC3314999

[24] Manber R, Bernert RA, Suh S, Nowakowski S, Siebern AT, Ong JC. CBT for Insomnia in patients with high and low depressive Symptom Severity: adherence and clinical outcomes. J Clin Sleep Med. 2011;07:645–52. 10.5664/jcsm.1472 10.5664/jcsm.1472 PMC3227711 22171204

[25] Peoples AR, Garland SN, Pigeon WR, Perlis ML, Wolf JR, Heffner KL, et al. Cognitive behavioral therapy for Insomnia reduces Depression in Cancer Survivors. J Clin Sleep Med. 2019;15:129–37. 10.5664/jcsm.7586 30621831 10.5664/jcsm.7586 PMC6329536

[26] Matthews EE, Berger AM, Schmiege SJ, Cook PF, McCarthy MS, Moore CM, et al. Cognitive behavioral therapy for Insomnia Outcomes in Women after primary breast Cancer Treatment: a Randomized, Controlled Trial. Oncol Nurs Forum. 2014;41:241–53. 10.1188/14.ONF.41-03AP 24650832 10.1188/14.ONF.41-03AP

[27] Espie C, Fleming L, Cassidy J, Samuel L, Taylor M, White L. Randomized Controlled Clinical Effectiveness Trial of Cognitive Behavior Therapy compared with treatment as Usual for Persistent Insomnia in patients with Cancer. J Clin Oncology: Official J Am Soc Clin Oncol. 2008;26:4651–8. 10.1200/JCO.2007.13.9006 10.1200/JCO.2007.13.9006 18591549

[28] Savard J, Simard S, Ivers H, Morin C. Randomized study on the efficacy of cognitive-behavioral therapy for Insomnia secondary to breast Cancer, part II: Immunologic Effects. J Clin Oncology: Official J Am Soc Clin Oncol. 2005;23:6097–106. 10.1200/JCO.2005.12.513 10.1200/JCO.2005.12.513 16135476

[29] Blom K, Jernelöv S, Rück C, Lindefors N, Kaldo V. Three-year Follow-Up of Insomnia and Hypnotics after Controlled Internet treatment for Insomnia. Sleep. 2016;39:1267–74. 10.5665/sleep.5850 27091535 10.5665/sleep.5850 PMC4863216

[30] van der Zweerde T, Bisdounis L, Kyle SD, Lancee J, van Straten A. Cognitive behavioral therapy for insomnia: a meta-analysis of long-term effects in controlled studies. Sleep Med Rev. 2019;48:101208. 10.1016/j.smrv.2019.08.002 31491656 10.1016/j.smrv.2019.08.002

[31] Garland SN, Carlson LE, Stephens AJ, Antle MC, Samuels C, Campbell TS. Mindfulness-based stress reduction compared with cognitive behavioral therapy for the treatment of Insomnia Comorbid with Cancer: a Randomized, partially blinded, Noninferiority Trial. JCO. 2014;32:449–57. 10.1200/JCO.2012.47.7265 10.1200/JCO.2012.47.7265 24395850

[32] Sivertsen B, Omvik S, Pallesen S, Bjorvatn B, Havik OE, Kvale G, et al. Cognitive behavioral therapy vs zopiclone for treatment of chronic primary insomnia in older adults: a Randomized Controlled Trial. JAMA. 2006;295:2851–8. 10.1001/jama.295.24.2851 16804151 10.1001/jama.295.24.2851

[33] Smith MT, Perlis ML, Park A, Smith MS, Pennington J, Giles DE, et al. Comparative Meta-analysis of Pharmacotherapy and Behavior Therapy for Persistent Insomnia. AJP. 2002;159:5–11. 10.1176/appi.ajp.159.1.5 10.1176/appi.ajp.159.1.5 11772681

[34] Haddad NE, Palesh O. Acupuncture in the treatment of Cancer-Related psychological symptoms. Integr Cancer Ther. 2014;13:371–85. 10.1177/1534735413520181 24501113 10.1177/1534735413520181

[35] Fiorentino L, McQuaid JR, Liu L, Natarajan L, He F, Cornejo M, et al. Individual cognitive behavioral therapy for insomnia in breast cancer survivors: a randomized controlled crossover pilot study. Nat Sci Sleep. 2010;2:1–8. 23616695 10.2147/NSS.S8004 PMC2953254

[36] Natsky AN, Vakulin A, Chai-Coetzer CL, Lack L, McEvoy RD, Lovato N, et al. Economic evaluation of cognitive behavioural therapy for insomnia (CBT-I) for improving health outcomes in adult populations: a systematic review. Sleep Med Rev. 2020;54:101351. 10.1016/j.smrv.2020.101351 32739824 10.1016/j.smrv.2020.101351

[37] Fleming L, MacMahon K. CBT-I in Cancer: we know it works, so why are we waiting? Curr Sleep Medicine Rep. 2015;1:177–83. 10.1007/s40675-015-0021-0

[38] Bower P, Gilbody S. Stepped care in psychological therapies: access, effectiveness and efficiency: narrative literature review. Br J Psychiatry. 2005;186:11–7. 10.1192/bjp.186.1.11 15630118 10.1192/bjp.186.1.11

[39] Ho FY-Y, Chung K-F, Yeung W-F, Ng TH, Kwan K-S, Yung K-P, et al. Self-help cognitive-behavioral therapy for insomnia: a meta-analysis of randomized controlled trials. Sleep Med Rev. 2015;19:17–28. 10.1016/j.smrv.2014.06.010 25104471 10.1016/j.smrv.2014.06.010

[40] Jernelöv S, Lekander M, Blom K, Rydh S, Ljótsson B, Axelsson J, et al. Efficacy of a behavioral self-help treatment with or without therapist guidance for co-morbid and primary insomnia -a randomized controlled trial. BMC Psychiatry. 2012;12:5. 10.1186/1471-244X-12-5 22264332 10.1186/1471-244X-12-5 PMC3328261

[41] Zachariae R, Amidi A, Damholdt MF, Clausen CDR, Dahlgaard J, Lord H, et al. Internet-delivered cognitive-behavioral therapy for insomnia in breast Cancer survivors: a Randomized Controlled Trial. J Natl Cancer Inst. 2018;110:880–7. 10.1093/jnci/djx293 29471478 10.1093/jnci/djx293 PMC6093474

[42] Koffel EA, Koffel JB, Gehrman PR. A meta-analysis of group cognitive behavioral therapy for insomnia. Sleep Med Rev. 2015;19:6–16. 10.1016/j.smrv.2014.05.001 24931811 10.1016/j.smrv.2014.05.001 PMC4910506

[43] Arem H, Lewin D, Cifu G, Bires J, Goldberg E, Kaltman R, et al. A feasibility study of Group-Delivered behavioral interventions for Insomnia among breast Cancer survivors: comparing cognitive behavioral therapy for Insomnia and a mind–body intervention. J Altern Complement Med. 2019;25:840–4. 10.1089/acm.2019.0038 31237434 10.1089/acm.2019.0038

[44] Zhou ES, Partridge AH, Syrjala KL, Michaud AL, Recklitis CJ. Evaluation and treatment of insomnia in adult cancer survivorship programs. J Cancer Surviv. 2017;11:74–9. 10.1007/s11764-016-0564-1 27495283 10.1007/s11764-016-0564-1 PMC5865603

[45] Johns MW. A New Method for Measuring Daytime Sleepiness: the Epworth Sleepiness Scale. Sleep. 1991;14:540–5. 10.1093/sleep/14.6.540 1798888 10.1093/sleep/14.6.540

[46] Chung F, Abdullah HR, Liao P, STOP-Bang Questionnaire. A practical Approach to screen for obstructive sleep apnea. Chest. 2016;149:631–8. 10.1378/chest.15-0903 26378880 10.1378/chest.15-0903

[47] Nagappa M, Liao P, Wong J, Auckley D, Ramachandran SK, Memtsoudis S, et al. Validation of the STOP-Bang Questionnaire as a Screening Tool for Obstructive Sleep Apnea among different populations: a systematic review and Meta-analysis. PLoS ONE. 2015;10:e0143697. 10.1371/journal.pone.0143697 26658438 10.1371/journal.pone.0143697 PMC4678295

[48] Brebach R, Sharpe L, Costa DSJ, Rhodes P, Butow P. Psychological intervention targeting distress for cancer patients: a meta-analytic study investigating uptake and adherence. Psycho-oncology. 2016;25:882–90. 10.1002/pon.4099 26893285 10.1002/pon.4099

[49] Vaughan E, Ftanou M, Lewin J, Murnane A, Berger I, Wiley JF, et al. AYA ‘Can-Sleep’ programme: protocol for a stepped-care, cognitive behavioural therapy-based approach to the management of sleep difficulties in adolescents and young adults with cancer. Pilot and Feasibility Studies. 2022;8:159. 10.1186/s40814-022-01128-7 35902975 10.1186/s40814-022-01128-7 PMC9331489

[50] Chevalier LL, Fine E, Sharma A, Zhou ES, Recklitis CJ. Evaluating the Sleep Treatment Education Program (STEP-1): a single-session educational workshop addressing insomnia in cancer survivors. J Psychosoc Oncol. 2023;41:123–32. 10.1080/07347332.2022.2054750 35468047 10.1080/07347332.2022.2054750

[51] Zhou ES, Recklitis CJ. Internet-delivered insomnia intervention improves sleep and quality of life for adolescent and young adult cancer survivors. Pediatr Blood Cancer. 2020;67:e28506. 10.1002/pbc.28506 32568460 10.1002/pbc.28506

